# Effectiveness of a campaign to implement chlorhexidine use for newborns in rural Haiti

**DOI:** 10.1186/s13104-017-3059-x

**Published:** 2017-12-19

**Authors:** Susan M. Walsh, Kathleen F. Norr, Heather Sipsma, Leslie A. Cordes, Girija Sankar

**Affiliations:** 1 Department of Women, Children, and Family Health Science, University of Illinois at Chicago, College of Nursing, 845 South Damen Avenue (M/C 802), Chicago, IL 60612 USA; 2 Benedictine University, 5700 College Road, Lisle, IL 60532 USA; 3 Northwestern University, Feinberg School of Medicine, 420 E Superior St, Chicago, IL 60611 USA; 4 The International Trachoma Initiative at the Task Force for Global Health, Atlanta, USA

**Keywords:** Behavior change campaign, Chlorhexidine, Umbilical cord, Neonate, Haiti

## Abstract

**Background:**

Chlorhexidine topical cord application is recommended to prevent umbilical cord infections in newborns delivered at home in low-resource settings. A community campaign introducing chlorhexidine for the first time in Haiti was developed. Traditional birth attendants (TBAs) were identified as implementers since they typically cut newborns’ cords. TBAs were trained to apply chlorhexidine to the cord and demonstrate this procedure to the mother. Concurrently TBAs explained reasons for using chlorhexidine exclusively instead of traditional cord care practices. The campaign’s effectiveness was evaluated 7–10 days post-delivery using a survey administered by community health workers (CHWs) to 198 mothers.

**Results:**

Nearly all mothers heard about chlorhexidine use and applied it as instructed. Most mothers did not initially report using traditional cord care practices. With further probing, the majority reported covering the cord but few applied an unhygienic substance. No serious cord infections were reported.

**Conclusion:**

The campaign was highly successful in reaching mothers and achieving chlorhexidine use. In this study, the concomitant use of traditional cloth coverings or bindings of the cord did not appear harmful; however more research is needed in this area. This campaign provides a model for implementing chlorhexidine use, especially where trained TBAs and CHWs are present.

## Background

Sepsis is the third leading cause of neonatal death worldwide [1], accounting for more than 520,000 deaths every year [2]. Although bacterial infection originating in the umbilical cord (omphalitis) is one of the leading cases of sepsis [3–6], these infections can be prevented by applying 7.1% (delivering 4%) chlorhexidine daily to the newborn’s umbilical stump during the first week of life [7–10]. Evidence suggests that home births in low income countries have an elevated risk for cord infection [8]. The World Health Organization (WHO) has consistently recommended the application of chlorhexidine to the umbilical cord stump of infants delivered in the community setting in low-resource countries since 1998 thus chlorhexidine remains on the WHO Model List of Essential Medicines [11–13]. However, the use of a topical antiseptic was also recommended if harmful traditional cord care practices included application of unhygienic substances or unclean cloth to the umbilical cord. An application of an antiseptic to the neonate’s cord per local preference was recommended by the WHO under such circumstances [11]. The WHO recognized that traditional practices were deeply rooted in local culture and intentions behind these practices were very complex. A variety of neonatal cord care practices in facilities and local communities across the globe were based on evidence from hospital nurseries in developed countries. Also concerns regarding procurement, cost and safe use of medications/antiseptics existed. These concerns along with the lack of a broader scope of research limited the advancement of furtherer evidence based cord care practice guidelines, especially in low-resource countries. In response to the WHO’s call to action, several reviews have sought evidence to support antiseptic use for neonatal cord care in both facility and in the community setting where unhygienic delivery is likely [8, 14, 15].

Despite these WHO recommendations, chlorhexidine 7.1% (delivering 4%) is not on the Haitian List of Essential Medicines. Thus chlorhexidine for cord care use has not been initiated in Haiti, among many other countries, where its benefits may be particularly important due to high rates of poverty, neonatal mortality and home deliveries. In Haiti, 62% of the population lives on less than $1.25 (US) a day, making Haiti the poorest country in the Western Hemisphere [16]. Haiti’s neonatal mortality rate is 25.5 deaths per 1000 live births, compared with less than 3 per 1000 in North America and 16.7 per 1000 in the Dominican Republic, Haiti’s island neighbor [16, 17]. In Haiti, neonatal mortality accounts for 37% of the under-five deaths, with 7% of these newborn deaths caused by sepsis [18]. In addition, 69% of neonates do not receive postnatal care within the first 2 days of birth [19]. Therefore interventions at birth are critical to reducing risk of infections among newborns in Haiti who are delivered at home. Although more than 90% of women in Haiti receive at least one antenatal visit, approximately 64% of deliveries occur at home [16].

One factor that complicates the use of chlorhexidine is the possibility that its use and/or effectiveness may be diminished by potentially harmful local practices regarding neonatal cord care [5, 20–22]. Studies in Bangladesh, Nepal, Pakistan, and Tanzania have identified potentially harmful traditional cord care practices [5–9, 23, 24] such as mustard oil applied to the neonates’ cords over 50% of the time in Sylhet District, Bangladesh [20]. Similar practices were reported in Petit-Goâve, Haiti, including the application of goat scat, dirt/dust, burnt nutmeg or cotton, crushed charcoal, ash, palm oil, and crushed leaves [25]. Another notable traditional cord care practice is to cover the cord site with cloth, gauze or abdominal bindings to prevent harm to the infant from “wind” (evil spirits) entering into the infant via the open umbilicus [25]. One study conducted in southern Nepal reported an incidence of cord infection of only 5.5%. However, when traditional substances were applied to the cord, the risk of neonatal cord infection increased to 29–62%, depending on the substance applied [5]. Cords that are not separated by day 5–7 can be an impetus for using traditional applications and/or coverings to prevent evil spirits from entering the infant via the open cord [25]. Although a number of studies are underway, there is not yet a well-established body of evidence regarding how concomitant use of traditional cord care practices affects chlorhexidine effectiveness.

In summary, chlorhexidine application is a simple, low-cost intervention to reduce neonatal cord infection. Potential acceptability of this new cord care practice among both mothers and community health workers in Haiti was previously demonstrated [25]; however, this evidence-based preventive measure had not been previously introduced in Haiti. To address this gap, a community behavior change campaign to introduce the application of chlorhexidine by new mothers in a rural area in Haiti was developed.

## Methods

This study had two primary aims:

1. To evaluate whether the campaign reached mothers delivering a baby at home in the target area; and
2. To determine how many mothers adhered to the instructions to apply chlorhexidine to the neonate’s cord site.

The study’s secondary aims were to identify whether mothers continued traditional cord care practices and whether there was any evidence that these practices decreased use of chlorhexidine or limited its effectiveness. The effect of maternal age, parity and living conditions on mothers’ use of chlorhexidine according to instructions and their use of traditional cord care practices was also explored.

A community behavior change campaign was developed to introduce chlorhexidine application in collaboration with Global Health Action (GHA), a non-profit organization that has been conducting community-based health and development programs in the district of Petit-Goâve Haiti since 1980 [26]. In this district of Haiti, 64 traditional birth attendants (TBAs) trained by GHA assist in approximately 900 home deliveries and post-partum/newborn visits annually. The 21 community health workers (CHWs) from GHA visit these newborns within 1 week, 1 month and 3 months post-delivery. The TBAs implemented the chlorhexidine umbilical cord care campaign and the CHWs collected data regarding mothers’ experiences with the campaign. After the campaign began, a brief survey was used with a cohort of the first 200 women delivered at home in the Petit-Goâve district, a procedure that avoided selection bias. Two of the mothers recruited by the CHWs were later identified as younger than 18 years old and were dropped from the sample leaving 198 mothers.

### Setting and sample

This study took place in Petit-Goâve, Haiti, a rural district with a population of 157,296, located 95 km from the capital, Port-Au-Prince [27]. Petit-Goâve is divided into thirteen communal sections. GHA trains and provides ongoing support to 64 TBAs and 21 CHWs in eight of these sections. TBAs routinely report each home birth they conduct.

Recruitment and enrollment of the mother occurred in the mother’s home 1 week post-delivery after the completion of the routine newborn visit by the CHW. Mothers were eligible if they were at least 18 years of age, experienced an uncomplicated, full term delivery as judged by the TBA’s report and the mother, and gave birth to an apparently healthy baby who was 7–10 days old. All women were checked for eligibility and invited to participate by the CHW following translated scripts in native Haitian Creole. Verbal consent was obtained by the CHW. Women who were not mentally or emotionally competent to respond to the questions were ineligible. None of the eligible mothers declined to participate. Universal sampling of all recently delivered women by GHA TBAs in the Petit-Goâve district was recruited. When a sample size of 200 eligible mother/infant dyads was reached recruitment was terminated.

### Design

The community campaign was developed in partnership with GHA, building upon GHA’s existing networks of trained TBAs and CHWs in Petit-Goâve. GHA provides ongoing support and monthly refresher training sessions for their TBAs and CHWs. TBAs assist in home-based deliveries in Petit-Goâve using clean delivery kits and routinely visit mothers and neonates the day after delivery. CHWs also provide ongoing health services in the community, including routine immunizations, micronutrient distribution, infant growth monitoring, and health education sessions on sanitation and hygiene promotion and nutrition [28].

The campaign aimed to teach mothers to replace the use of unhygienic substances with the application of chlorhexidine to their neonate’s cord. Mothers were also taught to leave the umbilical cord open to air with no coverings except typical clothing used for the neonate. The campaign was designed to follow the WHO’s recommendations for newborn umbilical cord care for this setting and incorporated the culturally unique findings regarding Haitian mothers’, TBAs’ and CHWs’ understanding of neonates susceptibility to illness [25]. Because TBAs are present at delivery and routinely cut the neonate’s cord, they were identified as the appropriate primary persons to implement the campaign; chlorhexidine application to the umbilical cord should be initiated within the first 24 h of life. To provide consistent messaging the local prenatal care nurse at a community clinic along with the CHWs were trained. This allowed mothers to learn about hygienic cord care and chlorhexidine application during prenatal care prior to delivery.

The campaign included three components. First, for prenatal sensitization, the community clinic nurse added a discussion of chlorhexidine as part of the prenatal care educational sessions, and posters about chlorhexidine application were placed in the clinic. Second, at each home birth, the TBA was instructed to tell the mother about the reasons to use chlorhexidine and apply chlorhexidine to the cord site while demonstrating the procedure to the mother. The last component was to give the mother a supply of chlorhexidine to use for 7 days or until the cord fell off. Chlorhexidine was not on Haiti’s Essential Medicines List and thus was unavailable to procure directly from a source in Haiti. GHA received a donated supply of Chlorxy-G gel tubes (25 g, delivering 4% chlorhexidine gluconate) from Drugfield Pharmaceuticals for study use only. As part of the campaign, the TBAs received a supply of chlorhexidine for their delivery kits. After demonstrating chlorhexidine application to the mother post-delivery, the TBA left the remainder of the chlorhexidine with the mothers.

The first step of the campaign was to design and translate the campaign messages and specific activities. The next step was to train local health workers to implement the campaign. There were two training sessions for TBAs and two for the CHWs, integrated into their regularly scheduled separate monthly meetings, which routinely include an educational component. The nurse at the community clinic attended all four training sessions as recognized health care leader within the Petit-Goâve district.

The first training session was the same for TBAs and CHWs and provided information, discussion, and then reinforcement about neonatal cord infections including probable causation, clinical manifestations, severity of these infections, safe application of chlorhexidine and our research protocol. The second training sessions differed for TBAs and CHWs. TBAs were specifically trained to apply chlorhexidine to the newborn’s umbilical stump shortly after cutting the cord. They were also trained to demonstrate this first application to the mother and instruct her to continue the application for 7 days or until cord separation, whichever occurred first. Cautions for proper use of chlorhexidine included hand washing prior to and after application and a warning to prevent eye contact with chlorhexidine. The facilitator demonstrated proper chlorhexidine application by using doll models, followed by peer reviewed return demonstrations by the TBAs using the doll models and role play. The session focused on appropriate dialogue between the TBA and the new mother at the time of delivery through role play and return demonstrations for chlorhexidine application. CHWs focused on how to consent mothers into the study and complete the survey, using demonstrations and peer reviewed demonstrations. Refresher training for the TBAs and CHWs occurred just prior to data collection and were repeated at regularly scheduled monthly meetings. Time for comments, questions and answers was provided throughout each phase of the training period. All training and refresher sessions were facilitated by GHA staff and an experienced Haitian medical doctor on the research team.

### Measures

All data came from the 1-week survey completed by the CHW after the regular visit at 1 week after birth. To evaluate the success of the campaign, we examined two primary outcomes, whether or not women received the message to use chlorhexidine [“Did anyone tell you anything about applying an antiseptic (chlorhexidine) to your baby’s cord”] and whether or not women adhered to the instructions to apply chlorhexidine for 7 days or until cord separation (if before 7 days). First, mothers were asked if they had heard of chlorhexidine and if so, who had told them about chlorhexidine. Second, those who had heard of chlorhexidine were asked if they had used the chlorhexidine antiseptic solution (yes/no); if they reported using chlorhexidine, they were then asked for how many days they applied the chlorhexidine. Women who reported applying chlorhexidine for fewer than 7 days were asked why they did not do so. Responses included “forgot”, “too difficult”, “did not like”, “was told not to do”, “baby’s cord fell off”, and “other”. We considered women who reported using chlorhexidine for all 7 days and women who reported using the chlorhexidine for fewer than 7 days but reported the reason for discontinuing application as “the baby’s cord fell off” as adhering to instructions for chlorhexidine application. Additional questions asked about the details of chlorhexidine application included the mothers’ source for information about chlorhexidine use, whether mothers found chlorhexidine easy or difficult to use, and how they remembered to apply it daily.

The use of traditional cord care practices was also assessed using a checklist of common practices identified in a study previously completed in Petit-Goâve, Haiti [25]. These practices were combined to create two yes/no indicators: use of gauze or cloth to cover the cord site and application of any substances other than chlorhexidine to the cord. A checklist of any symptoms that might indicate infant infection, completed based on both the CHW’s examination and the mother’s report, was used to create a dichotomous indicator of whether any symptoms were present. Demographic questions included age, parity, housing condition and religion.

### Procedure

After approval, GHA trained the TBAs, CHWs and the Olivier community nurse, and the TBAs received chlorhexidine for their birth kits. Using the list of recently delivered mothers from the TBAs’ reports; the CHWs then visited each mother’s house approximately 1 week after delivery. Following the CHW’s routine newborn assessment, the study was explained and informed consent was obtained from each participant after being given the opportunity to ask questions and/or opt out of participation. The CHW then completed a verbal questionnaire at 1 week post-delivery.

### Data analysis

Demographic variables were used to describe the sample and use of chlorhexidine. Unadjusted and adjusted analyses were used to determine factors related to adherence to the chlorhexidine cord application protocol and use of traditional cord care practices.

## Results

The mothers ranged in age from 18 to 43 (mean age 26.0 ± 5.3). The majority of mothers were in their 20s. Regarding parity, only 13.6% were first-time mothers, 66.2% of mothers were multiparas with 2–3 prior births and 20% were multiparas with 4 or more prior births (range 1–8). Almost 80% of mothers lived in potentially unhygienic delivery conditions as measured by having a dirt floor or living in a tent. When asked about their religion 57% of the mothers said they were Catholic, 34% said they were Protestant and 5% identified themselves as practicing Voudou (Table 1).

**Table 1:** Sample demographic characteristics (n = 198)

	n	%
Age of mother		
< 20	21	10.7
20–29	130	66.0
≥ 30	46	23.4
Parity		
Primiparas	27	13.6
Multiparas: 2–3 births	131	66.2
Multiparas: 4 or more births	40	20.2
Potentially unhygienic home	153	78.5
Religion^a^		
Catholic	110	57.3
Protestant	65	33.9
Voudou	10	5.2
Other	7	3.6

Missing values for each item range from 0 to 6

^a^Respondents were instructed to select all that apply

To evaluate the success of the campaign, the extent to which women received the message to use chlorhexidine and how many of them adhered to the instructions to apply for 7 days or until the cord fell off (if before 7 days) was examined. Only three of the 198 women (1.5%) said they had not been told to apply chlorhexidine to the baby’s cord. Of those who had been told to apply chlorhexidine, 98.4% of mothers reported that the TBA advised them about chlorhexidine use at the time of delivery and 8.4% were also told by the CHW. Most women had not heard about chlorhexidine use prenatally (Table 2). Adherence to the recommendation to apply chlorhexidine was also examined. Nearly all women (98%) applied chlorhexidine at least once. The number of days chlorhexidine was applied varied from 2 to 7 days, with 54% of mothers applying chlorhexidine for 7 days. Of those that did not apply chlorhexidine for 7 days, 81.5% reported that the baby’s cord had separated prior to day 7. Only 24% said that the cord fell off on day 7 or later. A total of 89% of mothers adhered to the instructions to apply for 7 days or until cord separation (Table 2).

**Table 2:** Use of chlorhexidine (n = 198)

	n	Valid%
Told about applying chlorhexidine to baby’s cord		
Yes	194	98.5
No	3	1.5
Among participants told about applying chlorhexidine		
Persons who advised about chlorhexidine use		
TBA	187	98.4
CHW	16	8.4
Doctor/nurse	1	0.5
Other	1	0.5
Used chlorhexidine		
Yes	190	98.4
No	3	1.6
Among participants who used chlorhexidine		
Number of days applied chlorhexidine		
1–2	3	1.7
3–4	37	20.4
5–6	43	23.7
7	98	54.1
Reasons for not applying chlorhexidine for all 7 days (among those who did not apply chlorhexidine for 7 days)		
Baby’s cord fell off	66	81.5
Forgot	1	1.2
Other	0	0
Among participants who reported the baby’s cord had fallen off		
Day baby’s cord fell off		
1–2	7	4.4
3–4	45	28.2
5–6	70	43.7
7 or later	38	23.8
Adhered to instructions^a^	164	88.6
Ease of chlorhexidine use		
Easy	178	94.2
Not too hard	11	5.8
Difficult	0	0

Numbers of missing responses per item range from 1 to 9

^a^A mother was considered adherent if she reported applying chlorhexidine for 7 days or if she reported that she had not completed the 7 days because the baby’s cord fell off; calculated among participants who were told about chlorhexidine (n = 185)

When asked about the ease of application, 94% of those who applied chlorhexidine said it was easy to use and 6% said it was not too hard to use. Only one mother reported forgetting to apply chlorhexidine. No one reported that the application was difficult to use, that they did not like the intervention, that they were told not to apply or mentioned any other reasons for not applying chlorhexidine (Table 2). When mothers were asked how they remembered to apply the chlorhexidine, the most common response was “diaper change”, mentioned by 66 mothers (38%). Other responses included, “on bedside table” by 11 (6.4%), at “wash time” by 13 (7.5%), when “breastfeeding” by three and “when I see the cord” was reported by five. Several mothers commented a desire to have chlorhexidine available at future deliveries.

Although the secondary aim of the campaign was to replace traditional cord care practices with chlorhexidine use, the majority of mothers said they still used traditional cord care practices. These included abdominal coverings (gauze, cloth, bindings) or applications of substances other than chlorhexidine. When mothers were first asked “Have you applied or done anything (else) to the baby’s umbilical cord over the past 7 days?”, only 8 out of 194 said they had. However, among the 120 mothers who were probed further, 79 (66%) said they had used coverings but only 4 (3%) said that they had put an unhygienic substance on the cord site which included powder, dirt, dung or water. Only one of these mothers applied dirt and no one applied dung (Table 3).

**Table 3:** Experiences with cord care

	n	Valid%
Substances applied to newborn cord (n = 120)		
None	30	25.0
Covering (cloth or binding)	79	65.8
Unhygienic substance^a^	4	3.3
Both covering or binding and an unhygienic substance	7	5.8
Symptoms observed in baby (n = 192)^b^		
Red cord	9	4.7
Swollen	2	1.0
Pus	10	5.2
Fever	1	0.5
Poor eating	0	0
Irritability	7	3.6
Any of these^c^	18	9.4
When you noticed these things, what did you do (n = 18)?^b^		
Contacted nurse/doctor/hospital	9	50.0
Contacted TBA/CHW	3	16.7
Contacted hogan	0	0

^a^Includes powder, dirt, dung or water

^b^Select all that applies

^c^Excludes irritability

There was no evidence that the concurrent use of traditional cord care practices had any relationship with the presence of symptoms in the infant. None of the newborns developed a serious illness and only 9% of mothers reported any mild to moderate symptom of infection in their newborn. The most common symptoms reported were pus, redness at the cord stump, and poor eating. Of the 9% who reported symptoms, most mothers took their infant to a health facility or provider and some consulted their TBA or CHW (Table 3).

The association between traditional cord care practices and symptoms of infection among mothers who provided data for each question (n = 116) was examined. Approximately 16% of mothers who did not use coverings or bindings reported one or more symptoms compared with 6% of mothers who used coverings only (Fisher’s exact test p > 0.05). Only one of the 10 mothers who reported using unhygienic substances (10%) reported that her infant had any symptoms that might indicate a cord infection compared with 8.5% of mothers who did not report using unhygienic substances (Fisher’s exact test p > 0.05). In this small sample size it appears that using coverings or applying substances while concurrently applying chlorhexidine to the cord site did not result in a higher rate of infection.

Living in unhygienic conditions (dirt floor or tent) related to infant symptoms was also explored. There were 188 mothers who answered both questions. Among the 148 mothers who lived in unhygienic conditions, 15 (10.1%) reported symptoms of infection. Among the 40 who indicated not living in unhygienic conditions, only 1 mother (2.5%) reported symptoms (Fisher’s exact test p > 0.05). Although only a minority of mothers reported living in hygienic conditions, these results suggest unhygienic conditions may be associated with the development of symptoms suggestive of cord infection.

The relationship between maternal age, parity and living in a potentially unhygienic home and adherence to chlorhexidine application and use of cord care coverings was examined (Table 4). No characteristics were significantly associated with adherence to chlorhexidine. Maternal age and parity, however, were significantly related to mothers’ use of coverings; the youngest mothers and those having their first babies were less likely to use coverings at the cord site compared with older mothers and those having their second or subsequent babies, respectively.

**Table 4:** Unadjusted associations between sociodemographic variables and adherence to instructions and whether or not participants used a binding on the newborn cord

	Adhered to instructions			Used covering		
	n (column%)		p value	n (column%)		p value
	Yes (n = 163)	No (n = 21)		Yes (n = 86)	No (n = 34)	
Mother’s age			0.595			0.026
< 20	20 (12.3)	1 (4.8)		7 (8.1)	8 (23.5)	
20–29	108 (66.3)	15 (71.4)		63 (73.3)	17 (50.0)	
≥ 30 years	35 (21.5)	5 (23.8)		16 (18.6)	9 (26.5)	
Parity			0.838			0.014
1st baby	23 (14.1)	3 (14.3)		6 (7.0)	9 (26.5)	
2nd or 3rd	108 (66.3)	15 (71.4)		62 (72.1)	20 (58.8)	
4th or higher	32 (19.6)	3 (14.3)		18 (20.9)	5 (14.7)	
Unhygienic home	128 (80.0)	15 (71.4)	0.365	60 (71.4)	29 (87.9)	0.061

## Discussion

Despite the proven efficacy of chlorhexidine application to prevent potentially life-threatening neonatal cord infections for infants born outside of health facilities in low-resource settings, no program to implement this intervention exists in Haiti. To fill this gap, we developed and evaluated a community behavior-change campaign for chlorhexidine use in a rural area of Haiti. The campaign was very successful in achieving its primary aims. After the campaign began, over 98% of mothers who gave birth at home in the target area had heard about the reasons to use chlorhexidine. Nearly all mothers adhered to the WHO recommended practice of applying chlorhexidine to the cord site and did not report any difficulties in doing so.

The campaign was not as successful in achieving its secondary aim of limiting potentially harmful traditional cord care practices. After the campaign was initiated, the majority of mothers continued to use coverings over the cord site. However, only a few mothers reported that they applied any unhygienic substance such as powder or dust to the cord site; no mother reported using dung. There was no indication that concurrent chlorhexidine use, cloth coverings/bindings or applying another substance to the cord site related to symptoms of neonatal infection. No infant had a serious cord infection or other illness. All of the infants in this study received at least an initial dose of chlorhexidine by the TBA when the cord was cut, and most also received daily application for 7 days or until the cord separated. Therefore these results should not be interpreted as supporting traditional cord care practices in the absence of chlorhexidine application.

This study provides strong evidence that trained TBAs are highly appropriate change agents to introduce the use of chlorhexidine for neonatal cord care in Haiti. Nearly all home births in Haiti are attended by a TBA, providing a viable mechanism for widespread implementation of chlorhexidine. The TBA is with the mother when the cord is cut, the exact time when chlorhexidine should be initiated. This provides a unique opportunity to apply the first dose while also role-modeling application for the mother. In previous studies mothers have shown concerns regarding delayed cord separation. Even if chlorhexidine might have caused a slight delay in cord separation, mothers continued daily chlorhexidine application, adding to evidence that chlorhexidine might be successfully introduced globally.

Future campaigns could be improved in several ways. Even though the TBAs were trained to teach mothers not to apply any other substance to their newborn’s cord, traditional coverings were not specifically discussed. Such discussions regarding cord site coverings would be beneficial. Future campaigns should also consider ways to improve mothers’ access to information prior to delivery. The campaign was designed to also provide information at the community clinic in the study area about exclusive chlorhexidine use. Most mothers did not attend that particular clinic and therefore did not receive these messages prior to birth. Although the single message from the TBA at the time of delivery resulted in chlorhexidine use by almost all mothers, this single message did not change the mother’s use of traditional cord coverings. Prenatal information would give mothers opportunities to plan ahead and consider how to address any difficulties they may encounter. This would be especially important in future roll-out when mothers may need to purchase chlorhexidine either prior to delivery or from TBAs at delivery. Providing information prenatally could also reach fathers, grandmothers and other support people, who can encourage mothers to apply chlorhexidine.

Global experts in the Healthy Newborn Network [29] have expressed concern that traditional cord care practices may discourage mothers from using chlorhexidine or limit its effectiveness. In this study, although most mothers continued to use a covering at the cord site, they also applied chlorhexidine as directed. Thus, for these mothers, traditional practices did not discourage chlorhexidine use. Also there was no evidence of harm related to traditional practices when concomitantly used with chlorhexidine application. There are strong beliefs in Haiti that covering the cord site is very important to protect the newborn [25]. It might be more culturally appropriate to provide clean and porous gauze to mothers at birth, thus respecting mothers’ beliefs while maintaining a clean and dry cord site. It is also important to note that younger mothers and first-time mothers were less likely to use coverings at the cord site, suggesting that there may be some decline in adherence to traditional cord care practices.

Data regarding chlorhexidine application, traditional cord care practices and symptoms of infection are all from maternal self-report which may be a limitation of this study. However, the CHW examined the neonate, providing independent corroboration of symptoms of infection at 1 week. In addition, probing for more detail about use of traditional cord care practices could be especially important as mothers were instructed by the TBAs in this study not to use these practices, thus mothers may have been reluctant to disclose such use to CHWs.

Transferability to other parts of Haiti may be another limitation to consider. GHA has a long history of working with TBAs and CHWs in the Petit-Goâve district. This campaign was able to build upon existing GHA resources, including a network of trained CHWs and TBAs, a regular meeting structure for training, and a high level of community trust in the TBAs and CHWs. This infrastructure is not present in all parts of Haiti, although there are other regions throughout the country where similar programs exist.

An important issue that needs to be dealt with at the national level in Haiti and other countries seeking chlorhexidine use is in-country availability of chlorhexidine. First, chlorhexidine 7.1% (delivering 4%) must be established on the country’s Essential Medicine List. Next, a sustainable procurement process must be established for a more widespread implementation of the WHO standards for neonatal cord care in low-resource settings and for eventual in-country scale up. A donated chlorhexidine supply specifically for this study was provided and mothers received a 7-day supply of chlorhexidine 7.1% (delivering 4%). In previous qualitative interviews with childbearing women in Haiti, women expressed willingness to purchase chlorhexidine if it was locally available and reasonably priced [25]. Further study is needed to identify how chlorhexidine could be distributed and at what cost so that pregnant women would be able to have an adequate supply at the time of delivery.

The next step to initiate chlorhexidine use throughout Haiti is to integrate the global context of newborn health into Haiti. The Millennium Development Goals data report can claim many successes; however the goal to decrease global neonatal mortality rate (NMR) was not met [30]. Both the WHO updated neonatal care protocols [31] and the United Nations’ Sustainable Development Goals [32] continue to focus efforts toward improving the healthy survival of newborns. As a response, low resource countries with a high NMR such as Haiti, must identify committed stakeholders and advocates from global, regional, country-specific and private sectors. Collaboration and varying forms of capacity strengthening will be needed in order to develop and initiate a framework for regional and country-specific chlorhexidine application programs.

A strategy for introducing chlorhexidine in the Latin American and Caribbean (LAC) region can be piloted in Haiti, the country with the highest NMR in the LAC region. At this time Haitian governance seeks to create an operational neonatal health plan to reduce morbidity and mortality as promoted by the LAC Neonatal Alliance [33]. The Helping Babies Survive and Thrive Program [34] is being promoted as a life-saving intervention in Haiti and the introduction of chlorhexidine would prove another valuable commodity by reducing neonatal deaths due to sepsis. Chlorhexidine 7.1% (delivering 4%) is one of four newborn lifesaving commodities, considered a country level systems indicator, and is recommended by the Partnership for Maternal, Newborn and Child Health [18, 35]. However the formulation of chlorhexidine 7.1% (delivering 4%) is not yet on Haiti’s Essential Medicines List [36]. Equally important is to identify a sustainable process to procure and deliver an available, affordable and sustainable chlorhexidine supply to Haiti.

## Conclusion

Findings suggest that mothers who delivered in their homes in Haiti served by regional TBAs will readily accept the use of chlorhexidine application for newborn cord care. To advance chlorhexidine use in Haiti technical assistance from the Chlorhexidine Working Group [29] would be helpful to address issues of cost and procurement, feasibility and sustainability. The Haiti Neonatal Alliance in conjunction with the Pan American Health Organization [37] are gateways to promoting chlorhexidine with the Haitian Ministry of Health (MSPP). Most countries with high NMRs recognize the impact of chlorhexidine use for cord care and will be able to promote its use if a clear country-specific strategy for introduction and scale up can be developed.

## Abbreviations

TBA: traditional birth attendant
CHW: community health worker
WHO: World Health Organization
GHA: Global Health Action
NMR: neonatal mortality rate
LAC: Latin American and Caribbean
MSPP: Haitian Ministry of Health
